# We should stop calling hysteroscopic tissue removal systems ‘morcellators’

**DOI:** 10.52054/FVVO.13.3.036

**Published:** 2021-09-24

**Authors:** Saridogan E

**Affiliations:** Birmingham Women’s Hospital; University College London, Institute for Women’s Health

The United Kingdom National Institute for Health and Care Excellence (NICE) released its guidance on ‘Hysteroscopic mechanical tissue removal (hysteroscopic morcellation) for uterine fibroids on 25 August 2021 (National Institute for Health and Care Excellence, 2021a). It concluded that evidence on the safety of hysteroscopic mechanical tissue removal (hysteroscopic morcellation) for uterine fibroids showed there were well recognised, infrequent but potentially serious side-effects. It stated that evidence on its efficacy was lim- ited in quantity and quality and that this procedure should only be used with special arrangements for clinical governance, consent, and audit or research. This means that clinicians using the procedure should collect data, for example by audit or research, and that risk of serious harm needs to be explained to the patient before they make a decision. It also requires that the clinical governance lead in their organisation is informed when this service is provided.

In contrast, a narrative review published in this issue of Facts, Views and Vision by Franchini et al. (2021) provides a comprehensive review of the available data on mechanical tissue removal systems (mHTR systems) and their performance against traditional hysteroscopic resection systems, and concludes that ‘gynaecologists should be encouraged to choose mHTR systems when available’.

The NICE recommendation came out in this format despite strong opposition from the national professional organisation, the British Society for Gynaecological Endoscopy (BSGE) (National Institute for Health and Care Excellence, 2021b). BSGE input highlighted that the published evidence in the NICE overview showed hysteroscopic morcellation of fibroids was safe and feasible for removing submucosal fibroids and pointed to better safety and ease of use compared to conventional electrical resection. According to the BSGE “it seemed a little perverse that hysteroscopic morcellation of fibroids is being singled out for these special arrangements and the need to inform clinical governance leads”.

As emphasised in the BSGE feedback the most serious risks associated with hysteroscopic myomectomy are uterine perforation and fluid overload and the prevalence of these complications are similar during mHTR and traditional electrical fibroid resection procedures. Hysteroscopic resection of fibroids has been in prac- tice since approximately 1985 and its safety and efficacy as a simpler alternative to abdominal myomectomy or hysterectomy have been widely accepted. However, hysteroscopic fibroid resection procedures require a high degree of surgical expertise for safe and complete removal of submucosal fibroids. As highlighted by Franchini et al (2021) comparative trials showed hysteroscopic morcellation technologies are easier to learn and are faster. In addition, mHTR systems eliminate the risk of diathermy damage in cases of uterine perfora- tion and avoid the need for wider cervical dilatation as they are thinner than the standard resectoscopes. As a result mHTR systems reduce ‘reliance on the surgeon’s operative skills’ and potentially improve the safety profile. Hence, this suggests that the NICE guideline which requires special arrangements on mHTR systems is counterproductive and may encourage clinicians and patients to choose a procedure (resection) that may have a slightly worse safety profile.

Historically, the NICE guidance on mHTR made an unfortunate start. In 2014, shortly after the release, the first guideline was quickly withdrawn at the height of the crisis on laparoscopic power morcellation, before the guideline was revised and reinstated in 2015. The current NICE guideline still states that there is a ‘theoretical risk of dissemination of malignancy with hysteroscopic morcellators’. This concern is somewhat related to the fact that similar terminology is used for ‘laparoscopic’ and ‘hysteroscopic’ morcellators. mHTR systems do not ‘morcellate’ the tissue. As Franchini et al. (2021) brings to our attention, the term originates from the French word ‘morcellement’ which means ‘to subdivide’. mHTRs do not subdivide the tissue but ‘shave’ and ‘aspirate’ it. Hence, ‘shaver’ is a better phrase for these devices. Perhaps the time has come to stop calling mHTR systems ‘morcellators’ and stop associating them with laparoscopic power morcellators.
